# Respiratory virus infections in symptomatic and asymptomatic children upon hospital admission: new insights

**DOI:** 10.1017/ash.2024.407

**Published:** 2024-10-07

**Authors:** Zachary M. Most, Trish M. Perl, Michael Sebert

**Affiliations:** 1 Division of Infectious Disease, Department of Pediatrics, University of Texas Southwestern Medical Center, Dallas, TX, USA; 2 Children’s Health System of Texas, Dallas, TX, USA; 3 Division of Infectious Diseases and Geographic Medicine, Department of Internal Medicine, University of Texas Southwestern Medical Center, Dallas, TX, USA; 4 Peter O’Donnell, Jr. School of Public Health, University of Texas Southwestern Medical Center, Dallas, TX, USA

## Abstract

**Objective:**

Describe and compare the prevalence of symptomatic and asymptomatic or recently resolved respiratory infections in hospitalized children.

**Design:**

Cross-sectional study.

**Setting:**

Three hospital primary-to-quaternary care pediatric healthcare system.

**Patients:**

People less than 22 years old who underwent admission screening for respiratory viruses using a multitarget polymerase chain reaction (PCR) panel from August 2020 through April 2022.

**Methods:**

The symptom status of each patient was recorded by the ordering provider. The prevalence of each virus was described comparing symptomatic and asymptomatic patients. Results for each virus were stratified by age group and trends were examined over time.

**Results:**

Of the 32,812 eligible PCR panels collected, 12,965 (39.5%), 18,651 (56.8%), and 1,196 (3.6%) were obtained from patients who were symptomatic, asymptomatic, or had missing or unknown symptom status, respectively. Symptomatic patients were much more likely to test positive for a respiratory virus (67.3% vs 27.0%). The most common viruses detected in asymptomatic patients were rhinovirus/enterovirus (18.0%), SARS-CoV-2 (3.6%), and parainfluenza viruses (2.3%). The odds ratio of testing positive when symptomatic was significantly greater than unity for all viruses but varied by virus and age group. The proportion of positive tests for each virus was dynamic and changed with intermittent epidemics, or viral “waves.”

**Conclusions:**

More than one-quarter of children without respiratory symptoms admitted to a pediatric healthcare system had PCR-detectable respiratory viruses. Children with symptoms of a respiratory infection are nevertheless much more likely to have a respiratory virus detected by PCR.

## Introduction

Respiratory viral infections (RVI) are ubiquitous, occur most frequently in children,^1–5^ and generate a large economic impact due to direct and indirect costs from missed days of work and school.^6–8^ RVIs can range in severity from asymptomatic infections to severe lower respiratory tract infections requiring hospitalization^9–13^ and are the leading cause of pediatric hospitalization in the USA.^14^ Respiratory viruses transmit easily from person to person by respiratory droplets, aerosolized particles, and contaminated surfaces.^15,16^ Therefore, it is not surprising that patients and healthcare personnel in pediatric hospitals are at risk for healthcare-associated RVI.^17–24^

Polymerase chain reaction (PCR) tests for respiratory viruses have excellent sensitivity and important clinical benefits.^25^ However, genetic material from respiratory viruses is commonly detected by nucleic acid amplification tests in asymptomatic adults and children,^2,26,27^ and infected individuals can have viral nucleic acids detectable by PCR for several weeks following an RVI.^28–30^ Hence, the positive predictive value of such PCR tests for an acute RVI has been called into question.^31–33^ The extent to which individuals with asymptomatic infections—or those still with PCR-detectable viral nucleic acids following resolution of an acute symptomatic infection—may pose a risk for secondary infections is unclear. Such transmission from asymptomatic individuals occurs with Severe Acute Respiratory Syndrome Coronavirus 2 (SARS-CoV-2) yet is less likely to occur than from symptomatic individuals.^34–38^

Prevention of healthcare-associated respiratory virus infections often focuses on interventions for symptomatic patients, or those who tested positive for a respiratory virus. To prevent transmission of respiratory viruses from asymptomatic patients, if it occurs, a better understanding of the prevalence of asymptomatic PCR-detectable RVIs in hospitalized patients is needed. Additionally, if asymptomatic PCR-detectable RVIs are common in certain pediatric populations, it is unclear whether symptomatic patients with positive PCR tests for a respiratory virus should necessarily have their symptoms attributed to that virus. We sought to compare the prevalence of PCR-detectable RVIs in asymptomatic versus symptomatic pediatric patients upon hospitalization.

## Methods

We conducted a retrospective cross-sectional study at a primary to quaternary care pediatric healthcare system that comprises three hospitals in North Texas, with over 30,000 hospital admissions per year. During much of the coronavirus disease 2019 (COVID-19) pandemic, all hospitalized patients were tested upon admission for SARS-CoV-2 infection from a nasopharyngeal swab regardless of the presence of symptoms. From August 2, 2020, to April 17, 2022, the recommended test at our facilities for patients upon admission, for indications other than elective surgeries, was a multitarget respiratory virus PCR panel (Biofire FilmArray RP 2.1, BioMérieux, Marcy-l’Étoile, France). This panel identified several bacterial pathogens and the following viruses: respiratory adenoviruses (ADV), common cold human coronaviruses (HKU1, NL63, 229E, or OC43 [ccCoV]), human metapneumovirus (hMPV), influenza A (H1N1pdm09 or H3N2 [FluA]), influenza B (FluB), parainfluenza (types 1-4 [PIV]), respiratory syncytial virus (RSV), rhinovirus/enterovirus (which are not distinguished on the panel [REV]), and SARS-CoV-2.

Demographic data, respiratory panel results, location of testing, hospitalization status, and symptom status were collected for all children, adolescents, and young adults aged 0 through 21 years (inclusive) who presented to any of the hospitals within the system and were admitted to an inpatient or observation unit. The first respiratory panel collected on the calendar day of admission, +/–1 calendar day, for each admission was included. All other tests for respiratory viruses, including single-target SARS-CoV-2 tests (which were used to screen surgical patients before inpatient or ambulatory procedures) were excluded. Individuals were excluded if they were not admitted to the hospital, had their tests collected two or more calendar days before or after the day of admission, or were 22 years old or older on the day of test collection. Repeat tests on the same individual during the same encounter were not included in the analysis.

A prevalent RVI was defined as having a positive test on the respiratory panel for one or more viruses. Age was categorized into the following: 0–11 months, 1–4 years, 5–9 years, 10–14 years, and 15–21 years. Symptom status was determined by the provider who ordered the respiratory panel in an electronic order-set using the Epic electronic health record (Epic Systems Corporation, Verona, WI; Supplemental Methods). The order-set asked the user if the patient had symptoms of COVID-19 using the phrase “Is the patient symptomatic as defined by CDC.” The user could select “Yes,” “No,” or “Unknown.” Those with unknown or missing symptom status were excluded from the analysis. The Centers for Disease Control and Prevention (CDC) definition of COVID-19 symptoms included: fever or chills, cough, shortness of breath or difficulty breathing, fatigue, muscle or body aches, headache, new loss of taste or smell, sore throat, congestion or runny nose, nausea or vomiting, and diarrhea.

The accuracy of the symptom status designation as applied to respiratory symptoms or fever was validated by a chart review of 50 randomly selected patients; 10 from each of five hospital/unit strata (Hospital A, B, or C and acute care services or intensive care unit, one hospital did not have an intensive care unit). We assessed this using positive and negative predictive value with the reviewer designation (ZMM) as the reference standard. Additionally, we performed a qualitative assessment to explore reasons for non-agreement.

The number of tests collected over time and the proportion positive for each virus, stratified by symptom status, were compared between symptomatic and asymptomatic patients with the chi-squared test and an odds ratio (OR) and 95% confidence interval (CI). We evaluated interaction with age by calculating the odds ratios and 95% CI for each virus and each age group. We used the chi-squared test for heterogeneity to evaluate interaction with age for each virus. We also compared the proportion positive for each virus by calendar week compared between symptomatic and asymptomatic patients.

This study was reviewed by the institutional review board at UT Southwestern Medical Center and was exempted as non-regulated research.

## Results

**Figure 1.**
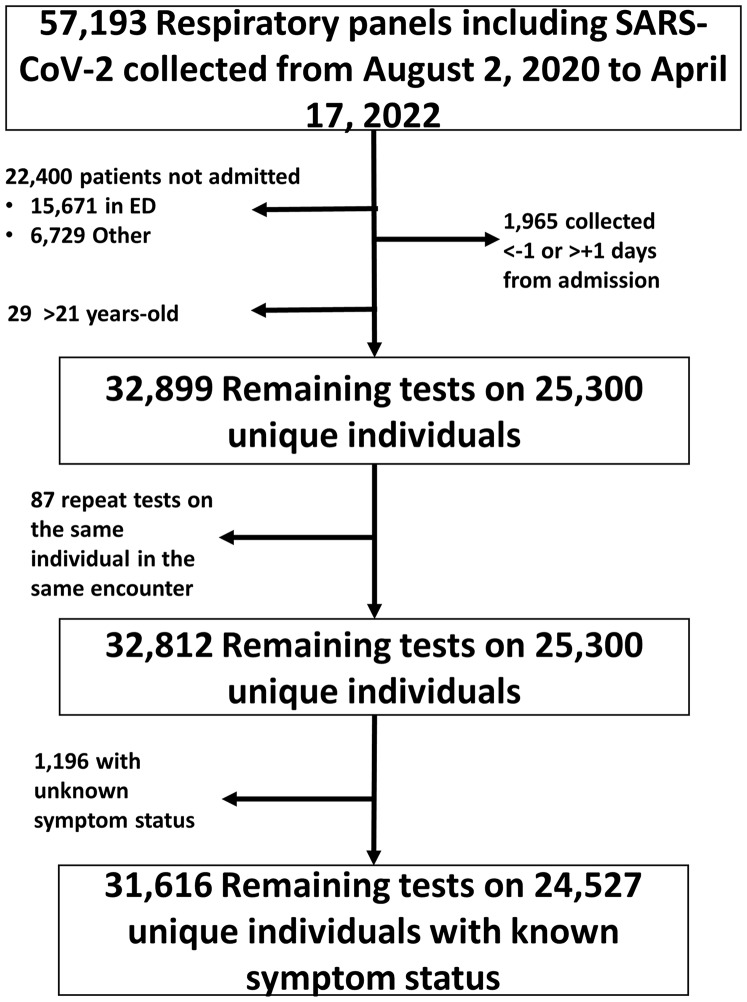
Flow diagram for inclusion of respiratory panels. SARS-CoV-2, severe acute respiratory syndrome coronavirus 2; ED, emergency department.

Between August 2, 2020, and April 17, 2022, 57,193 respiratory panels were collected (Supplemental Figure 1), of which 32,812 panels (averaging 52.6 panels per day) on 25,300 unique patients were included (Figure 1). Of these patients, 18,651 (56.8%) were asymptomatic, 12,965 (39.5%) were symptomatic, and 1,196 (3.7%) had unknown symptom status. The median age of patients tested was 4 years (IQR 1 to 12 years) with a slight predominance of males (53.3%). Most tests were on patients who were admitted to acute care services (95.2%) and a smaller number on those admitted to an intensive care unit (4.1%). The majority (89.7%) of tests were collected on the same calendar day as admission, with 10.3% collected the calendar day after admission and 0.03% collected the calendar day before admission (Table 1).

**Table 1.** Demographic and testing characteristics for 32,812 individuals admitted to the hospital with respiratory panels collected

Characteristic	Category	Total n = 32,812 (%)	Asymptomatic n = 18,651 (%)	Symptomatic n = 12,965 (%)	Unknown Symptom Status n = 1,196 (%)
Age	0–11 months	7,279 (22.2)	3,471 (18.6)	3,525 (27.2)	283 (23.7)
	1–4 years	9,270 (28.3)	3,757 (20.1)	5,198 (40.1)	315 (26.3)
	5–9 years	5,546 (16.9)	3,329 (17.9)	2,026 (15.6)	191 (16.0)
	10–14 years	5,819 (17.7)	4,338 (23.3)	1,255 (9.7)	226 (18.9)
	15–21 years	4,898 (14.9)	3,756 (20.1)	961 (7.4)	181 (15.1)
Age, median (IQR), years		4 (1–12)	8 (1–14)	2 (0–6)	5 (1–12)
Sex	Female	15,320 (46.7)	9,102 (48.8)	5,668 (43.7)	550 (46.0)
	Male	17,483 (53.3)	9,544 (51.2)	7,294 (56.3)	645 (53.9)
	Other/Unknown	9 (0.0)	5 (0.0)	3 (0.0)	1 (0.1)
Acuity of Admission^a^	Acute care	31,233 (95.2)	17,740 (95.1)	12,419 (95.8)	1,074 (89.8)
	Critical care	1,328 (4.1)	691 (3.7)	527 (4.1)	110 (9.2)
	Other/Unknown	251 (0.8)	220 (1.2)	19 (0.2)	12 (1.0)
Hospital of Admission	Hospital A	24,801 (75.6)	15,035 (80.6)	8,770 (67.6)	996 (83.3)
	Hospital B	7,786 (23.7)	3,406 (18.3)	4,191 (32.3)	189 (15.8)
	Hospital C	225 (0.7)	210 (1.1)	4 (0.0)	11 (0.9)
Days from admission to test collection^b^	–1 calendar day	11 (0.0)	6 (0.0)	5 (0.0)	0 (0.0)
	0 calendar days	29,428 (89.7)	16,533 (88.6)	11,794 (91.0)	1,101 (92.1)
	1 calendar day	3,373 (10.3)	2,112 (11.3)	1,166 (9.0)	95 (7.9)

IQR, interquartile range.

a Critical care included pediatric intensive care unit, neonatal intensive care unit, and cardiac intensive care unit.

b Date of test collection minus date of admission.

For a random sample of 50 cases, the overall agreement between the ordering provider and the reviewer was 80%. The positive predictive value was 93.3%, and the negative predictive value was 74.3%. Among patients marked as symptomatic by the ordering provider there was disagreement with the reviewer in one out of 15 cases (6.7%). Among patients marked as asymptomatic by the ordering provider there was disagreement with the reviewer in nine out of 35 cases (25.7%). Most discrepancies were due to the presence of respiratory symptoms that were mild in comparison to the indication for admission or were explained by an alternate etiology (Supplemental Results).

**Figure 2.**
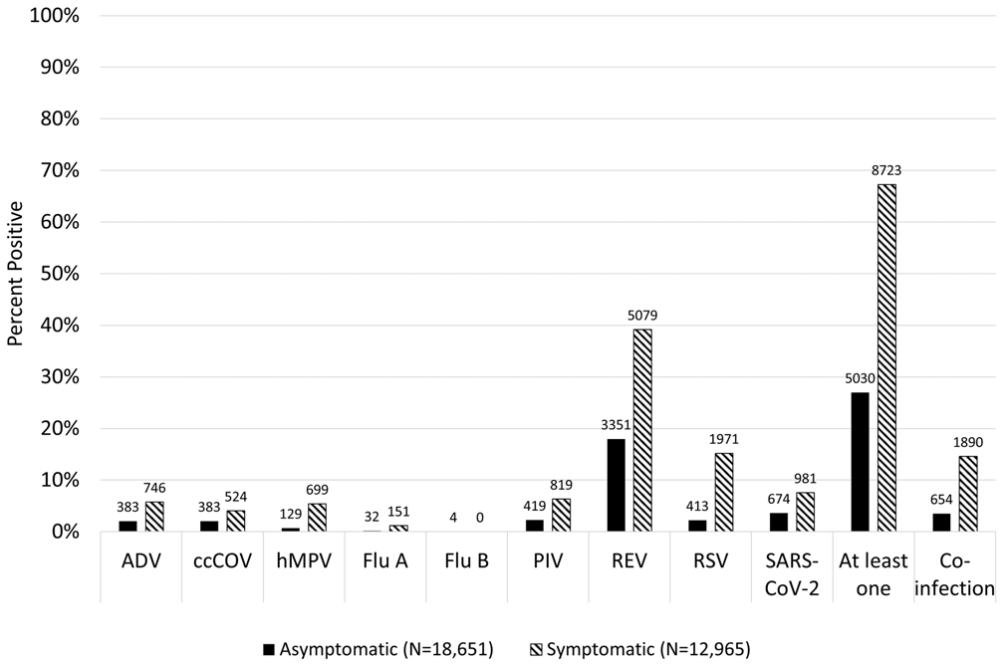
Proportion of respiratory viral polymerase chain reaction tests positive for each of 9 viruses upon hospital admission for asymptomatic and symptomatic individuals. ADV, respiratory adenoviruses; ccCOV, common cold coronaviruses; hMPV, human metapneumovirus; Flu A, influenza A; Flu B, influenza B; PIV, parainfluenza viruses; REV, rhinovirus/enterovirus; RSV, respiratory syncytial virus; SARS-CoV-2, severe acute respiratory syndrome coronavirus 2.

Among the 12,965 symptomatic patients and the 18,651 asymptomatic patients, at least one respiratory virus was identified in 67.3% and 27.0% of patients, respectively. Rhinovirus/enterovirus was the most commonly detected virus in both symptomatic and asymptomatic patients (39.2% and 18.0% positive, respectively). Among symptomatic patients, the next most-commonly detected viruses were RSV (15.2%), SARS-CoV-2 (7.6%), and parainfluenza viruses (6.3%). Among asymptomatic patients the next most-commonly detected viruses were SARS-CoV-2 (3.6%), parainfluenza viruses (2.3%), and RSV (2.2%). Viral coinfections were detected in 14.6% of symptomatic patients, and in 3.5% of asymptomatic patients (Figure 2).

There were differences in the proportion of positive tests by age group. For example, for rhinovirus/enterovirus, the proportion of tests that were positive for symptomatic and asymptomatic patients was greater for preschool-aged (1–4 years old) and early school-aged (5–9 years old) children than for older children and infants. Across all age groups, symptomatic patients were more likely to test positive for rhinovirus/enterovirus than asymptomatic patients. There were similar findings for other viruses (Supplemental Table).

**Figure 3.**
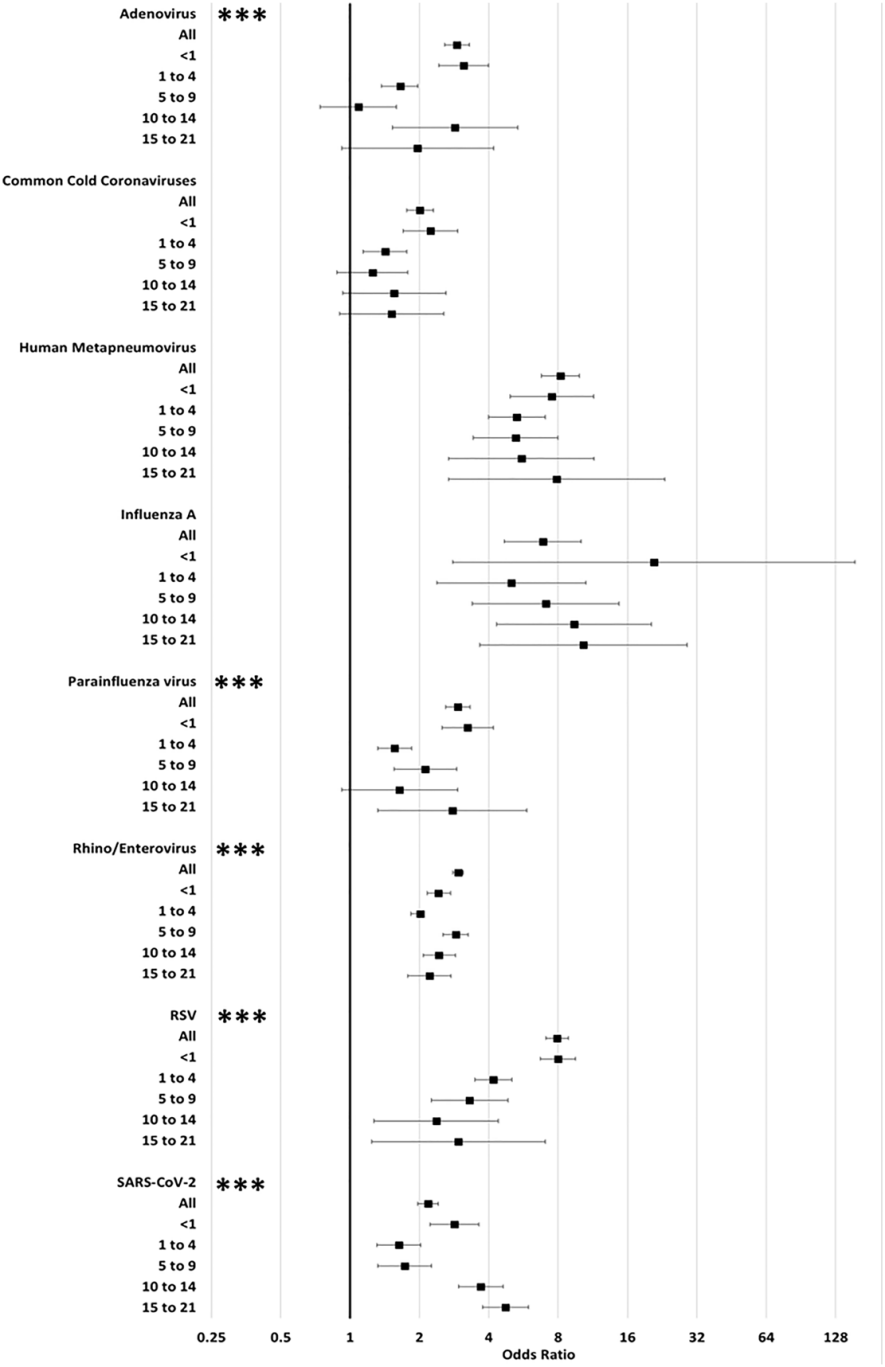
Odds ratio for prevalent respiratory virus infection comparing symptomatic to asymptomatic individuals for each virus, stratified by age group. Each virus was tested for interaction between symptom status and age with the chi-squared test for heterogeneity. *** indicates the *P* value for interaction was less than 0.001.

The odds ratio of testing positive when symptomatic compared to asymptomatic was significantly greater than unity for all viruses (Figure 3). It was greatest for hMPV (OR 8.18, 95% CI [6.76–9.91]), and it was lowest for ccCoV (OR 2.01, 95% CI [1.76–2.30]). There was strong evidence of interaction between age group and symptomatology for REV, ADV, PIV, RSV, and SARS-CoV-2, whereas there was no evidence of interaction between age group and symptomatology for ccCoV, hMPV, and FluA (Figure 3). There were only four infections with FluB detected during the study period, all in asymptomatic patients. For rhinovirus/enterovirus the OR for a positive test among symptomatic patients was lower for preschool-aged children than for school-aged children. For SARS-CoV-2 the corresponding OR was lowest for 1–4 and 5–9-year-olds, and greater for infants and older children/adolescents. For adenovirus the OR was greatest for infants, was near unity for 5–9-year-olds, and had more imprecise estimates due to fewer events in older children/adolescents. For RSV, the OR was greatest for infants. For ccCoV, the OR was low in all age groups.

**Figure 4.**
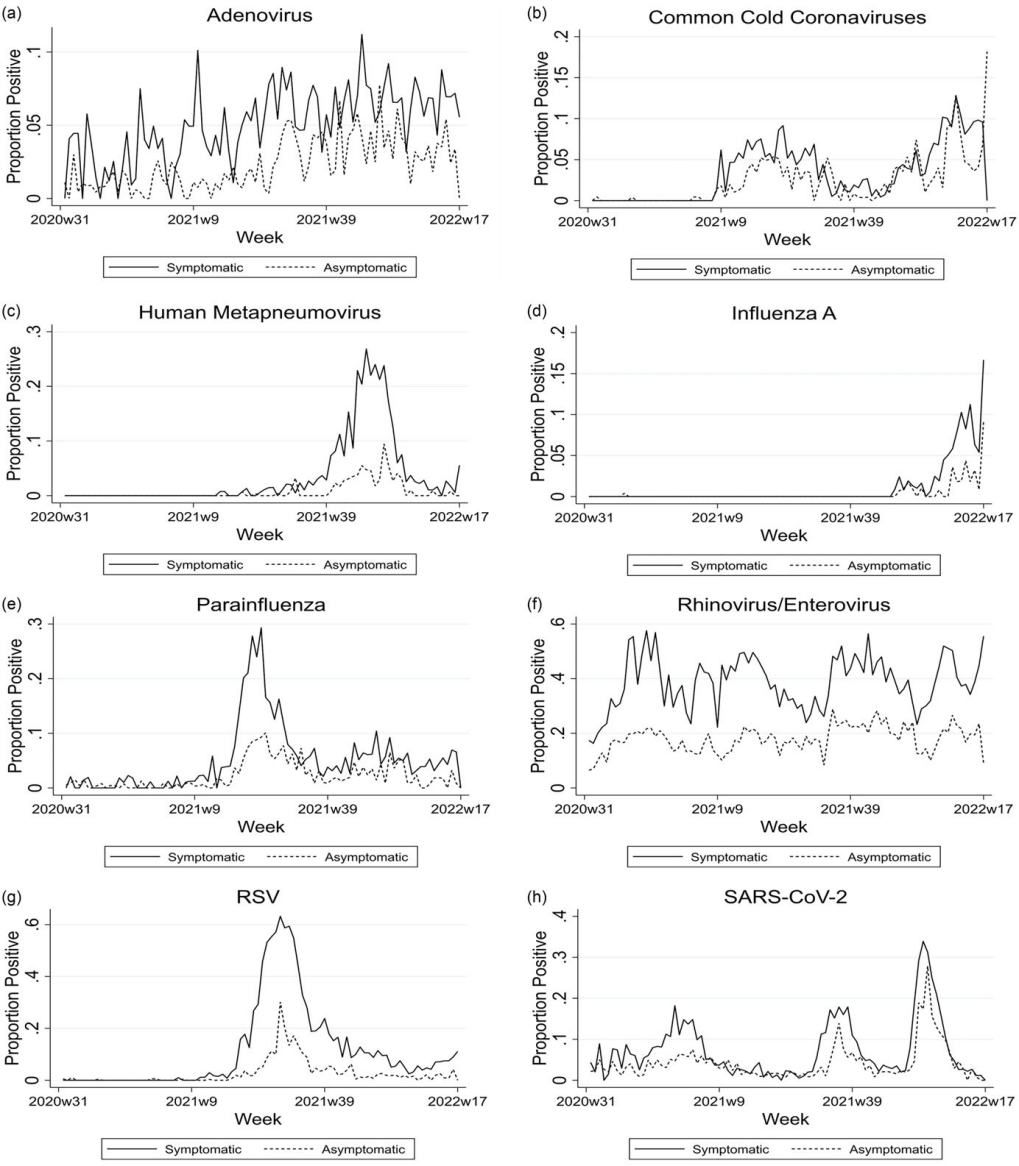
a–h Proportion of polymerase chain reaction tests positive for each virus upon hospital admission for asymptomatic and symptomatic individuals each week over time. RSV, respiratory syncytial virus.

The proportion of positive tests for each virus was dynamic and changed with seasonal epidemics or viral “waves.” Additionally, the ratio of positive test proportions between symptomatic and asymptomatic patients varied over time. Early in each SARS-CoV-2 “wave,” the proportion of tests that were positive rose more quickly for symptomatic than for asymptomatic patients. By the middle of each wave the positive test proportion for asymptomatic patients rose sharply and reached as high as 25% during the initial omicron wave in the winter of 2021–22. During the period between waves asymptomatic patients were often as likely to test positive for SARS-CoV-2 as symptomatic ones. For some other viruses (REV, hMPV, and RSV) there was always a substantial gap between the positive test proportion in symptomatic and asymptomatic patients (Figure 4a–h).

## Discussion

In this large, 21-month, retrospective study, respiratory viruses were commonly detected in asymptomatic patients hospitalized at a pediatric healthcare system. We report the dynamic epidemiology of respiratory viruses from a period during the SARS-CoV-2 pandemic when universal screening of admissions was mandated, providing a unique opportunity for study. All viruses were detected significantly more often in symptomatic patients, suggesting that symptoms may be attributed to the detected virus for many patients with positive PCR results. Variation among different viruses and by patient age, however, was important. RSV and influenza A were the most likely infections to present with respiratory symptoms, especially in infants. For several virus/age-group pairs the presence of symptoms did not significantly impact the likelihood of a positive PCR, including ADV in 5–9-year-olds and ccCoV in 5–21-year-olds. This suggests that for these virus/age-group pairs, a positive test in a symptomatic patient is as likely to be the result of incidental carriage as it is to represent the actual etiology of the patient’s symptoms.

The relationship between symptomatic and asymptomatic carriage also varied by virus over time. For SARS-CoV-2 there tended to be a large gap between the proportion of positive tests in symptomatic and asymptomatic patients during each “wave” that persisted until near the end of the “wave.” However, during the low prevalence periods between “waves” there was little difference in test positivity among those with and without symptoms. Not all viruses displayed this pattern. For example, for RSV, which had an uncharacteristic epidemic in the summer of 2021, a large gap between positive test proportions in symptomatic and asymptomatic patients was identified throughout the entire epidemic and continued thereafter. Additionally, for REV, which circulated throughout the entire study period, there was always a large gap between the proportions of positive tests in the symptomatic and asymptomatic patients.

This study shows clearly that patients who are asymptomatic with PCR-detectable respiratory viruses are frequently encountered in children’s hospitals. However, it remains unclear to what extent these children may be infectious and hence would pose a risk for secondary transmission. A recent study demonstrated that such asymptomatic transmission is rare under some conditions in healthcare settings.^38^ It is also unknown how many of these children had symptomatically recovered from recent viral infections and how many truly had asymptomatic or pauci-symptomatic cases. This is an area that requires further investigation to answer these questions.

This study has several strengths. This study was done during a period where testing was mandated, which captured almost all non-elective admissions and limited testing bias. Compared to other studies that have investigated the prevalence of asymptomatic respiratory infections in children,^2,26,27^ this study was much larger with over 5,000 PCR-detectable infections in children characterized as asymptomatic upon admission. The number of patients afforded the unique opportunity to compare the differences between viruses and between pediatric age-groups. Additionally, this study was able to evaluate the change in the proportion of positive tests over time, which revealed that the relationship between asymptomatic and symptomatic infections is not constant. Finally, by focusing on individuals who were admitted to pediatric hospitals, this study investigated a population that has substantial importance to understanding infection prevention efforts.

The study was limited by challenges inherent in correctly classifying symptom status. For asymptomatic patients who tested positive for a virus, we were unable to differentiate individuals who were truly asymptomatic (or pauci-symptomatic) from those who recently recovered from a symptomatic infection. The presence of symptoms was determined by the ordering provider who classified the patient based on symptoms seen with COVID-19. These symptoms included headache and diarrhea, which are atypical features of the more localized respiratory infections caused by many other viruses. The validation of symptom classification demonstrated that the degree of agreement between the ordering provider and the presence of respiratory symptoms or fever upon chart review was moderate. Most discrepancies occurred in patients marked as asymptomatic by the ordering provider. As a result, some patients labeled asymptomatic in our data set may have been misclassified and actually had symptoms of a respiratory infection. This would bias our results toward a higher asymptomatic prevalence. From the hospital infection prevention perspective, however, it may nonetheless be meaningful that the providers classified these patients as asymptomatic. If providers viewed some patients at a high level as asymptomatic despite mild respiratory symptoms that may not have been related to the reason for admission, infection prevention interventions targeted toward symptomatic patients may be less effective. Additionally, the study population only included individuals being admitted to a pediatric hospital, so the results may not be generalizable to children in the community or child-care settings.

In conclusion, we found that PCR-detectable respiratory viruses are common in asymptomatic children admitted to the hospital. Children with symptoms of a respiratory infection were nevertheless much more likely to have a respiratory virus detected. Further studies are needed to determine if these asymptomatic patients pose a risk for nosocomial transmission of respiratory viruses, and if such transmission can be mitigated by admission screening and/or use of barrier precautions.

## Supporting information

Most et al. supplementary material Most et al. supplementary material

## Supplementary material

For supplementary material accompanying this paper visit http://doi.org/10.1017/ash.2024.407. click here to view supplementary material
