# Supplementary material for: Respiratory virus infections in symptomatic and asymptomatic children upon hospital admission: new insights
Source: Antimicrob Steward Healthc Epidemiol. 2024 Oct 7;4(1):e162. doi: 10.1017/ash.2024.407 (PMC11474761; doi:10.1017/ash.2024.407)
Supplement: Most et al. supplementary material [file S2732494X24004078sup001.docx]

Supplement to: Respiratory virus infections in symptomatic and asymptomatic children upon hospital admission: new insights

# Supplemental Methods

Screenshot of Logic in Electronic Health Record: Symptom status was determined by the ordering provider in an electronic order-set under the category “Symptomatic as defined by CDC?” with the choices: Yes, No, Unknown


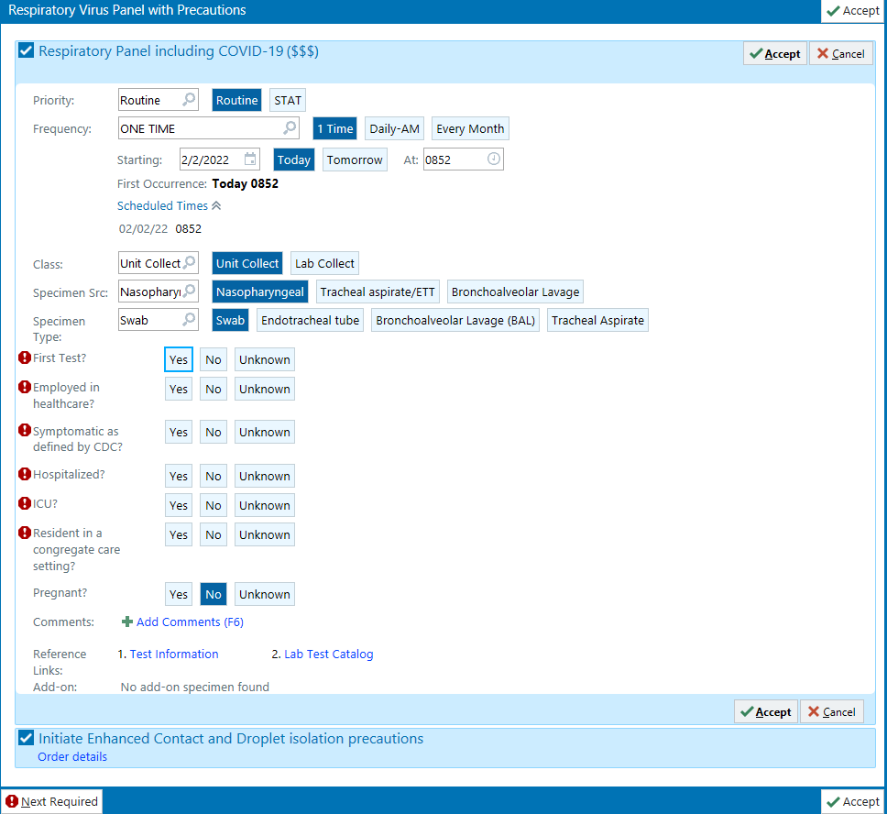


This screenshot from Epic demonstrates the logic and refers to the CDC definition of COVID-19 symptoms, which include: fever or chills, cough, shortness of breath or difficulty breathing, fatigue, muscle or body aches, headache new loss of taste or smell, sore throat, congestion or runny nose, nausea or vomiting, diarrhea. Earlier versions of this order set labeled this question “Is the patient symptomatic (respiratory or fever without source identified)?”

# Supplemental Results

## Supplemental Figure 1: Number of respiratory viral panels performed by month; August 2020-April 2022.


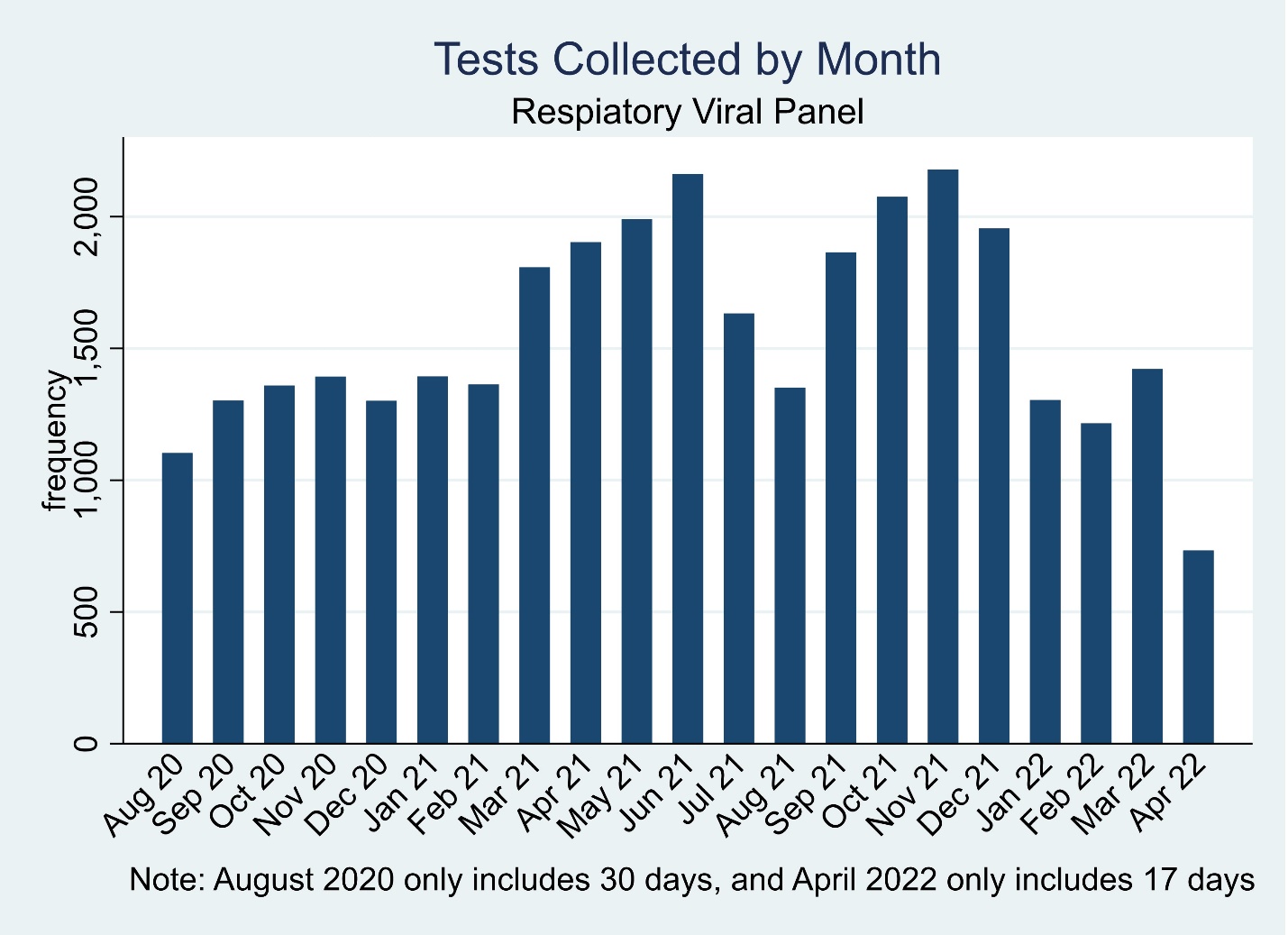


## Validation of symptom status

10 randomly selected patients from each of 5 hospital/unit strata (50 selected in total) had charts reviewed to determine the presence of fever or respiratory symptoms (cough, shortness of breath, congestion, or sore throat) when presenting to medical care (completed by ZMM). Validity of symptom status selected by the ordering provider was measured compared to the reviewer’s designation as the reference standard.

|  |  | Reviewer | |  |
| --- | --- | --- | --- | --- |
|  |  | Symptomatic (respiratory or fever) | Asymptomatic (respiratory or fever) | Total |
| Ordering  provider | Symptomatic as defined by CDC | 14 | 1 | 15 |
|  | Asymptomatic as defined by CDC | 9 | 26 | 35 |
|  | Total | 23 | 27 | 50 |

Of the 23 patients labeled symptomatic by the reviewer, 14 were identified as symptomatic by the ordering provider (Sensitivity = 60.9%). Of the 27 patients labeled asymptomatic by the reviewer, 26 were identified as asymptomatic by the ordering provider (Specificity = 96.3%). Of the 15 patients who were identified as symptomatic by the ordering provider, 14 were labeled symptomatic by the reviewer (Positive Predictive Value = 93.3%). Of the 35 patients who were identified as asymptomatic by the ordering, 26 were labeled asymptomatic by the reviewer (Negative Predictive Value = 74.3%). On qualitative review, two of these nine (22%) discrepancies were because patients had respiratory symptoms but had an alternative attributable cause (shortness of breath due to acidosis, or cough due to feeding intolerance) and four (44%) were due to symptoms that were mild and were not related to the indication for admission. Only three of the discrepancies had no explanation.

In an alternate analysis, the reviewer compared the reported symptom status to the presence of any symptoms of COVID-19 as defined by CDC. This was what the actual order the users interacted with had asked for and was broader than just respiratory symptoms and fever (included fever or chills, cough, shortness of breath or difficulty breathing, fatigue, muscle or body aches, headache new loss of taste or smell, sore throat, congestion or runny nose, nausea or vomiting, diarrhea).

|  |  | Reviewer | |  |
| --- | --- | --- | --- | --- |
|  |  | Symptomatic as defined by CDC | Asymptomatic as defined by CDC | Total |
| Ordering  provider | Symptomatic as defined by CDC | 15 | 0 | 15 |
|  | Asymptomatic as defined by CDC | 16 | 19 | 35 |
|  | Total | 31 | 19 | 50 |

In this case, the Positive Predictive Value was 100% but the Negative Predictive Value was poorer (52.3%). This was mostly due to the presence of gastrointestinal symptoms in patients who were identified as asymptomatic by the ordering provider.

|  | Table. Proportion of positive tests in symptomatic and asymptomatic individuals by age group for each virus | | | | |
| --- | --- | --- | --- | --- | --- |
| Virus | Age group |  | No. Tests | No. Positive | Percent Positive |
| REV |  |  |  |  |  |
|  | 0-11 months | Asymptomatic | 3471 | 589 | 17.0% |
|  | 0-11 months | Symptomatic | 3525 | 1168 | 33.1% |
|  | 1-4 years | Asymptomatic | 3757 | 1192 | 31.7% |
|  | 1-4 years | Symptomatic | 5198 | 2514 | 48.4% |
|  | 5-9 years | Asymptomatic | 3329 | 771 | 23.2% |
|  | 5-9 years | Symptomatic | 2026 | 940 | 46.4% |
|  | 10-14 years | Asymptomatic | 4338 | 517 | 11.9% |
|  | 10-14 years | Symptomatic | 1255 | 311 | 24.8% |
|  | 15-21 years | Asymptomatic | 3756 | 282 | 7.5% |
|  | 15-21 years | Symptomatic | 961 | 146 | 15.2% |
|  |  |  |  |  |  |
| ADV |  |  |  |  |  |
|  | 0-11 months | Asymptomatic | 3471 | 89 | 2.6% |
|  | 0-11 months | Symptomatic | 3525 | 267 | 7.6% |
|  | 1-4 years | Asymptomatic | 3757 | 184 | 4.9% |
|  | 1-4 years | Symptomatic | 5198 | 406 | 7.8% |
|  | 5-9 years | Asymptomatic | 3329 | 68 | 2.0% |
|  | 5-9 years | Symptomatic | 2026 | 45 | 2.2% |
|  | 10-14 years | Asymptomatic | 4338 | 22 | 0.5% |
|  | 10-14 years | Symptomatic | 1255 | 18 | 1.4% |
|  | 15-21 years | Asymptomatic | 3756 | 20 | 0.5% |
|  | 15-21 years | Symptomatic | 961 | 10 | 1.0% |
|  |  |  |  |  |  |
| ccCOV |  |  |  |  |  |
|  | 0-11 months | Asymptomatic | 3471 | 78 | 2.2% |
|  | 0-11 months | Symptomatic | 3525 | 172 | 4.9% |
|  | 1-4 years | Asymptomatic | 3757 | 132 | 3.5% |
|  | 1-4 years | Symptomatic | 5198 | 255 | 4.9% |
|  | 5-9 years | Asymptomatic | 3329 | 74 | 2.2% |
|  | 5-9 years | Symptomatic | 2026 | 56 | 2.8% |
|  | 10-14 years | Asymptomatic | 4338 | 47 | 1.1% |
|  | 10-14 years | Symptomatic | 1255 | 21 | 1.7% |
|  | 15-21 years | Asymptomatic | 3756 | 52 | 1.4% |
|  | 15-21 years | Symptomatic | 961 | 20 | 2.1% |
|  |  |  |  |  |  |
| hMPV |  |  |  |  |  |
|  | 0-11 months | Asymptomatic | 3471 | 26 | 0.7% |
|  | 0-11 months | Symptomatic | 3525 | 189 | 5.4% |
|  | 1-4 years | Asymptomatic | 3757 | 57 | 1.5% |
|  | 1-4 years | Symptomatic | 5198 | 392 | 7.5% |
|  | 5-9 years | Asymptomatic | 3329 | 29 | 0.9% |
|  | 5-9 years | Symptomatic | 2026 | 89 | 4.4% |
|  | 10-14 years | Asymptomatic | 4338 | 12 | 0.3% |
|  | 10-14 years | Symptomatic | 1255 | 19 | 1.5% |
|  | 15-21 years | Asymptomatic | 3756 | 5 | 0.1% |
|  | 15-21 years | Symptomatic | 961 | 10 | 1.0% |
|  |  |  |  |  |  |
| Flu A |  |  |  |  |  |
|  | 0-11 months | Asymptomatic | 3471 | 1 | 0.0% |
|  | 0-11 months | Symptomatic | 3524 | 21 | 0.6% |
|  | 1-4 years | Asymptomatic | 3756 | 8 | 0.2% |
|  | 1-4 years | Symptomatic | 5196 | 55 | 1.1% |
|  | 5-9 years | Asymptomatic | 3329 | 9 | 0.3% |
|  | 5-9 years | Symptomatic | 2023 | 38 | 1.9% |
|  | 10-14 years | Asymptomatic | 4336 | 9 | 0.2% |
|  | 10-14 years | Symptomatic | 1255 | 24 | 1.9% |
|  | 15-21 years | Asymptomatic | 3756 | 5 | 0.1% |
|  | 15-21 years | Symptomatic | 961 | 13 | 1.4% |
|  |  |  |  |  |  |
| Flu B |  |  |  |  |  |
|  | 0-11 months | Asymptomatic | 3471 | 1 | 0.0% |
|  | 0-11 months | Symptomatic | 3525 | 0 | 0.0% |
|  | 1-4 years | Asymptomatic | 3757 | 0 | 0.0% |
|  | 1-4 years | Symptomatic | 5198 | 0 | 0.0% |
|  | 5-9 years | Asymptomatic | 3329 | 1 | 0.0% |
|  | 5-9 years | Symptomatic | 2026 | 0 | 0.0% |
|  | 10-14 years | Asymptomatic | 4338 | 1 | 0.0% |
|  | 10-14 years | Symptomatic | 1255 | 0 | 0.0% |
|  | 15-21 years | Asymptomatic | 3756 | 1 | 0.0% |
|  | 15-21 years | Symptomatic | 961 | 0 | 0.0% |
|  |  |  |  |  |  |
| PIV |  |  |  |  |  |
|  | 0-11 months | Asymptomatic | 3471 | 82 | 2.4% |
|  | 0-11 months | Symptomatic | 3525 | 256 | 7.3% |
|  | 1-4 years | Asymptomatic | 3757 | 211 | 5.6% |
|  | 1-4 years | Symptomatic | 5198 | 442 | 8.5% |
|  | 5-9 years | Asymptomatic | 3329 | 73 | 2.2% |
|  | 5-9 years | Symptomatic | 2026 | 92 | 4.5% |
|  | 10-14 years | Asymptomatic | 4338 | 36 | 0.8% |
|  | 10-14 years | Symptomatic | 1255 | 17 | 1.4% |
|  | 15-21 years | Asymptomatic | 3756 | 17 | 0.5% |
|  | 15-21 years | Symptomatic | 961 | 12 | 1.2% |
|  |  |  |  |  |  |
| RSV |  |  |  |  |  |
|  | 0-11 months | Asymptomatic | 3471 | 187 | 5.4% |
|  | 0-11 months | Symptomatic | 3525 | 1103 | 31.3% |
|  | 1-4 years | Asymptomatic | 3757 | 148 | 3.9% |
|  | 1-4 years | Symptomatic | 5198 | 762 | 14.7% |
|  | 5-9 years | Asymptomatic | 3329 | 41 | 1.2% |
|  | 5-9 years | Symptomatic | 2026 | 80 | 3.9% |
|  | 10-14 years | Asymptomatic | 4338 | 25 | 0.6% |
|  | 10-14 years | Symptomatic | 1255 | 17 | 1.4% |
|  | 15-21 years | Asymptomatic | 3756 | 12 | 0.3% |
|  | 15-21 years | Symptomatic | 961 | 9 | 0.9% |
|  |  |  |  |  |  |
| SARS-CoV-2 |  |  |  |  |  |
|  | 0-11 months | Asymptomatic | 3471 | 91 | 2.6% |
|  | 0-11 months | Symptomatic | 3525 | 250 | 7.1% |
|  | 1-4 years | Asymptomatic | 3757 | 121 | 3.2% |
|  | 1-4 years | Symptomatic | 5198 | 267 | 5.1% |
|  | 5-9 years | Asymptomatic | 3329 | 112 | 3.4% |
|  | 5-9 years | Symptomatic | 2026 | 115 | 5.7% |
|  | 10-14 years | Asymptomatic | 4338 | 180 | 4.1% |
|  | 10-14 years | Symptomatic | 1255 | 173 | 13.8% |
|  | 15-21 years | Asymptomatic | 3756 | 170 | 4.5% |
|  | 15-21 years | Symptomatic | 961 | 176 | 18.3% |
